# The Semi-Centennial of the Discovery of Anesthesia

**Published:** 1896-12

**Authors:** 


### EDITORIAL.

The office of the Business Manager of The Journal is Nos. 311 and 312 Fitten Building.

The editors are not responsible for views expressed by contributors.

Articles for publication should reach this office not later than the 15th inst.

Address all communications, and make all remittances payable to The Atlanta Medical and Surgical Journal, Atlanta, Ga.

Reprints of articles will be furnished, when desired, at a nominal cost. Requests for the same should always be made *on the manuscript*.

IF A SUBSCRIBER NOTIFIES US TO STOP HIS JOURNAL AND IT IS NOT DONE, OR, IF HE ORDERS HIS ADDRESS CHANGED AND IT IS NOT, HE MAY KNOW THAT WE DID NOT GET HIS ORDER. MOVING TO ANOTHER ADDRESS WITHOUT NOTIFYING US, DOES NOT EXCUSE HIM FROM PAYING HIS BILL.

### THE SEMI-CENTENNIAL OF THE DISCOVERY OF ANESTHESIA.

We give space this month to a report of the late celebration at the Massachusetts General Hospital of the semi-centenial of the discovery of anesthesia. In this connection, also, we are glad to be able to be able to publish the excellent article by Dr. Roswell Park on the history and introduction of anesthesia. The nature of this article justifies us in calling particular attention to it. It was evidently written from a desire to be absolutely fair to all the parties to the ether controversy. He does not advocate anybody's claim—there is no necessity for that any longer—he has reviewed and presented the essential history of the use and introduction of ether as an anesthetic, and his statement of these circumstances is by far the best that we have ever seen. With the history of the introduction of anesthesia it is every medical man's duty to be familiar. We especially commend the paper of Dr. Park as worthy of careful reading.

